# The alpha7-nicotinic receptor contributes to gp120-induced neurotoxicity: implications in HIV-associated neurocognitive disorders

**DOI:** 10.1038/s41598-018-20271-x

**Published:** 2018-01-29

**Authors:** Coral M. Capó-Vélez, Bryan Morales-Vargas, Aurian García-González, José G. Grajales-Reyes, Manuel Delgado-Vélez, Bismark Madera, Carlos A. Báez-Pagán, Orestes Quesada, José A. Lasalde-Dominicci

**Affiliations:** 1 University of Puerto Rico, Río Piedras Campus, Department of Biology, San Juan, P.R 00931-3360 Puerto Rico; 2 University of Puerto Rico, Río Piedras Campus, Department of Physical Sciences, San Juan, P.R 00931-3360 Puerto Rico; 3 University of Puerto Rico, Río Piedras Campus, Department of Chemistry, San Juan, P.R 00931-3360 Puerto Rico; 4 University of Puerto Rico, Molecular Sciences and Research Center, San Juan, P.R 00926 Puerto Rico

## Abstract

Currently, there are no specific therapies to treat HIV-1 associated neurocognitive disorders (HAND). The HIV-1 envelope, gp120, induces neuropathological changes similar to those in HAND patients; furthermore, it triggers an upregulation of the α7-nicotinic acetylcholine receptor (α7-nAChR), facilitating intracellular calcium overload and neuronal cell death. Using a gp120_IIIB_-transgenic mouse (gp120-tgm) model, we demonstrate that α7-nAChRs are upregulated on striatal neurons. Activation of α7-nAChRs leads to an increase in both intracellular calcium and percentage of apoptotic cells, which can be abrogated by antagonizing the receptor, suggesting a role for α7-nAChRs in gp120-induced neurotoxicity. Moreover, we demonstrate for the first time that gp120-tgm have learning deficiencies on a striatum-dependent behavioral task. They also show locomotor deficiencies, which improved with α7-nAChR antagonists, further supporting a role for this receptor in gp120-induced neurotoxicity. Together, these results uncover a new mechanism through which gp120-induced modulation of α7-nAChRs in the striatum can contribute to HAND development.

## Introduction

HIV-associated neurocognitive disorders (HAND), a spectrum of disorders that range from asymptomatic cognitive impairment to severe dementia, remain a serious complication arising from HIV-1 infection despite treatment with combined antiretroviral therapy^1–3^. The onset of the most severe manifestation of the disease, HIV-associated dementia, which correlates with high plasma viral loads, has decreased due to effective virologic control. However, the development of minor neurocognitive disorders or asymptomatic neurocognitive disorders, milder forms of HAND, are much more prevalent. Approximately 50% of HIV^+^ individuals can develop some cognitive impairment that affects psychomotor functions, learning, and executive functionality, among others^2,4,5^. Thus, it is evident that studies focused on identifying new targets to develop appropriate treatment regimens for HAND are imperative.

Further understanding of HAND neuropathogenesis and the development of novel therapeutic approaches greatly benefits from the use of appropriate animal models. Specifically, the gp120_IIIB_-transgenic mouse (gp120-tgm) model developed by Toggas *et al*.^6^, recapitulates several of the abnormalities seen in the brain of HIV^+^ individuals including reactive microgliosis, astrocytosis and neuronal loss, neuropathological changes, age-dependent cognitive decline in spatial memory reference, and impaired proliferation and differentiation of neural progenitor cells^3,6–8^. This type of model is useful because it provides information about the effects of gp120 in the brain^3^ at cellular, molecular, cognitive, and locomotor levels.

In 2012, Ballester *et al*. identified the α7-nicotinic acetylcholine receptor (α7-nAChR), one of the most common cholinergic receptors in the mammalian brain, as an unrecognized contributor in HIV-induced neurotoxicity^9^. Using both *in vitro* and *in vivo* models, it was demonstrated that gp120_IIIB_ induces an upregulation of the α7-nAChR^9–11^. In fact, in the *in vitro* model, this functional upregulation facilitates elevated calcium entry and promotes neuronal cell death^9^. In the *in vivo* model (gp120-tgm), molecular evidence revealed a spatial difference in α7-nAChR expression within brain regions. Specifically, the basal ganglia’s primary input, the striatum, showed a sustained increase in α7-nAChR expression^9^. These results are consistent with the mild cognitive impairment exhibited by HAND patients, which stem from basal ganglia-associated injuries^12,13^ and a large accumulation of gp120 in the basal ganglia^14^. However, no study has focused on striatum-dependent cognitive and/or motor deficiencies due to gp120 exposure, even though this region is highly affected in HAND patients^13,15–17^.

Herein, we addressed the involvement of α7-nAChRs on gp120-induced neurotoxicity using an *in vivo* model. We aimed to determine α7-nAChR’s contribution to CNS degeneration in HIV-infected patients, focusing on the striatum of the gp120-tgm. We also implemented an experimental strategy to evaluate locomotor activity, a striatum-dependent behavioral component previously unexamined in this model, using an activity wheel device. Moreover, a pharmacological approach to assess the functionality of α7-nAChRs was also examined with bupropion, an FDA-approved drug and noncompetitive antagonist of α7-nAChRs^18^, and methyllycaconitine (MLA), a competitive antagonist of this cholinergic receptor^19^.

These results are of particular interest because they provide substantial knowledge about the gp120-induced neurotoxicity in the brain based on the expression, regulation, and activation of the α7-nAChR. Due to its pivotal role on cognition processes and the fact that it is expressed not only in the brain but also in a wide variety of immune cells targeted during HIV infection, the α7-nAChR could emerge as a pharmacological intervention target to develop neuroprotective therapeutics for HIV-infected patients suffering from HAND.

## Results

### The α7-nAChR is upregulated on striatal neurons isolated from gp120-tgm

**Figure 1:**
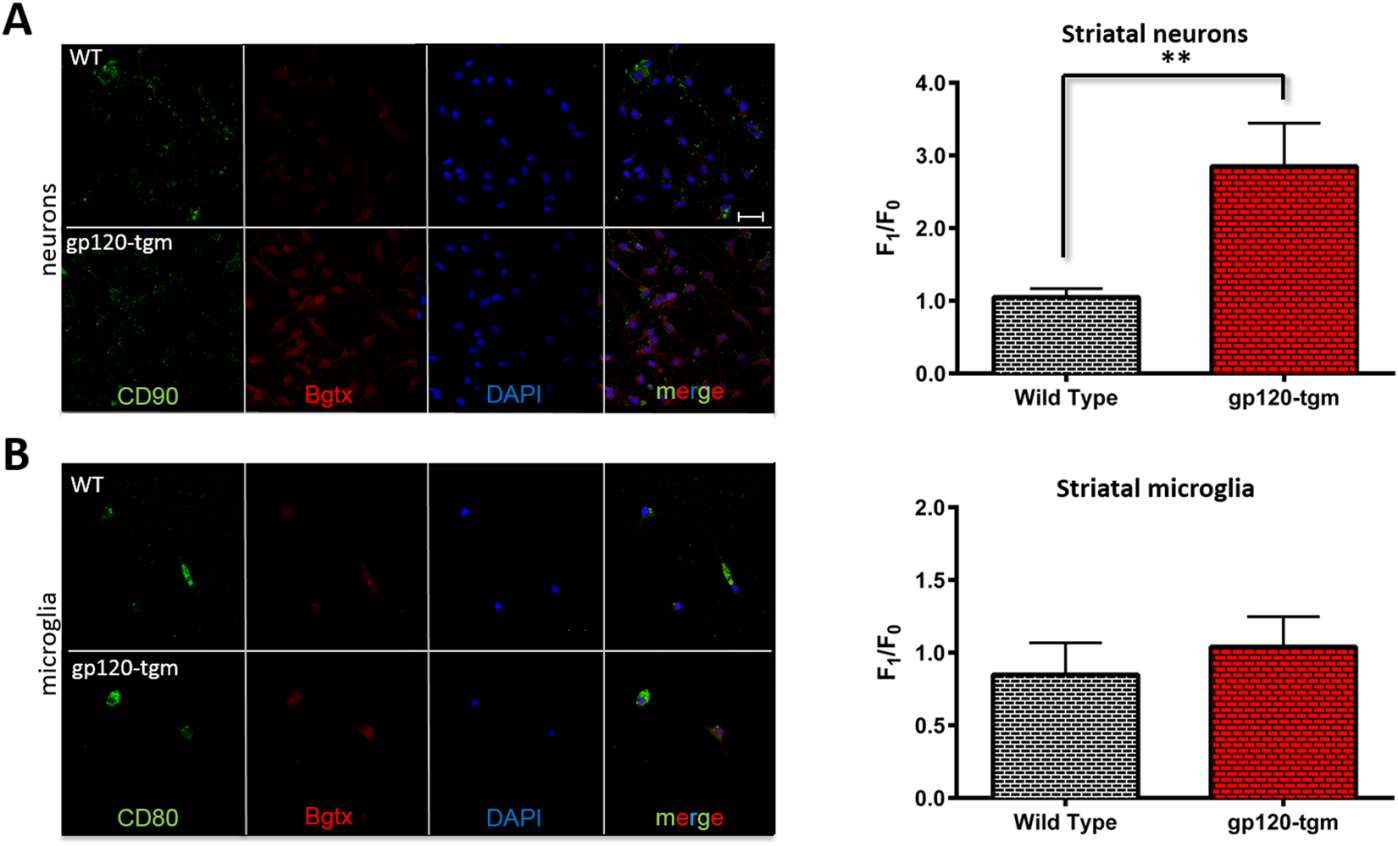
The α7-nAChR is upregulated on neurons, but not microglia, isolated from the striatum of gp120-tgm. Striatal neurons and microglia were isolated from WT and gp120-tgm adult mice’s brain. Cells were incubated with (**A**) CD90 (neuronal marker – green) or (**B**) CD80 (microglia marker - green), Bgtx Alexa 555 (α7-nAChR marker – red), and DAPI (nucleus marker – blue), to then measure fluorescence levels on a confocal microscope (40x). (**A**) Results demonstrate an increase in Bgtx binding, which is limited to neurons (*n* = 5 mice/strain), indicating an increase in α7-nAChR expression levels. Quantification of fluorescence shows a 3-fold increase in α7-nAChR expression on striatal neurons from gp120-tgm mice. (**B**) Microglia (*n* = 3 mice/strain) also express α7-nAChR but do not deviate significantly on fluorescence intensity. Results are shown as mean ± SEM values. Unpaired Student’s *t*-test, **P ≤ 0.01. Scale bar = 40 μm for all images in panels A and B.

It has been demonstrated that in our gp120-tgm model, the α7-nAChR is upregulated in the striatum, as evidenced by quantitative RT-PCR and immunoblot studies^9^. Because the α7-nAChR is present on neurons and microglia, we sought to investigate which of these cells is responsible for its increased expression within the gp120-tgm striatum. Striatal neurons and microglia from wild type (WT) and gp120-tgm were isolated, cultured, and incubated with a fluorescently-labeled α7-nAChR antagonist, α-bungarotoxin (Bgtx)^20^, which allowed us to make a relative quantification of receptor expression using confocal imaging. Consistent with Ballester *et al*.^9^, results show that α7-nAChR is upregulated in the striatum (Fig. 1A), as evidenced by an increase in Bgtx binding. However, this upregulation is not seen on microglia (Fig. 1B), it is only observed in striatal neurons (Fig. 1A). Gp120-tgm striatal neurons exhibited a 3-fold increase in α7-nAChR expression levels as compared to WT neurons (Fig. 1A; *P* ≤ 0.01). To confirm this region specificity, we also examined neurons and microglia from the hippocampus of WT and gp120-tgm and found no significant differences in α7-nAChR expression (Supplemental Fig. 1). Again, these results are consistent with Ballester *et al*.^9^ findings, where the α7-nAChR upregulation is restricted to the striatum, and suggest that not only is the α7-nAChR upregulation region-specific, but also cell-type specific. Because only striatal neurons exhibited α7-nAChR upregulation, they were the focus of subsequent experiments.

### Gp120-induced α7-nAChR upregulation depends on CXCR4 activation

**Figure 2:**
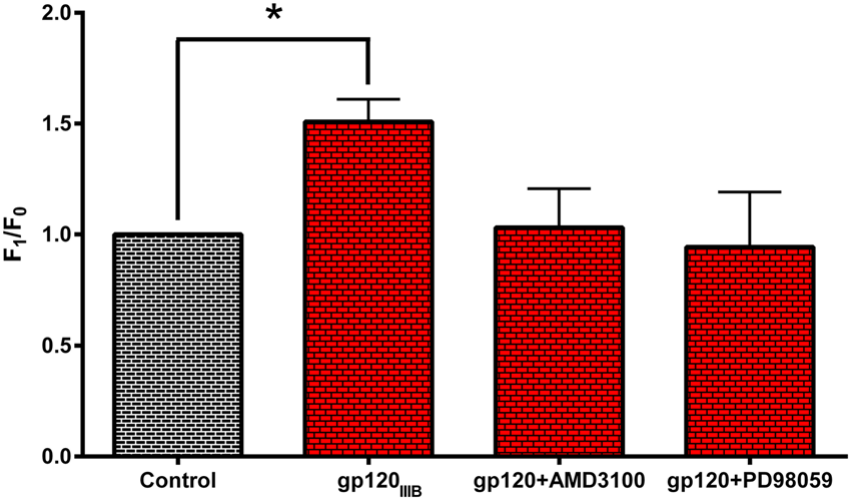
CXCR4 mediates the effects of gp120 on striatal neurons. Striatal neurons isolated from WT mice were treated with: 1) gp120_IIIB_, 2) gp120_IIIB_ + AMD3100, or 3) gp120_IIIB_ + PD98059 for 48 hrs. Then, cells were incubated with Alexa Fluor 555 α-bungarotoxin to measure α7-nAChR expression levels by confocal imaging (20x). Treatment with gp120_IIIB_ induced an α7-nAChR upregulation on striatal neurons, which is abolished by pretreatment with a CXCR4 antagonist (AMD3100), suggesting that this upregulation is CXCR4-dependent. Additionally, blocking the Ras-Raf-MEK pathway (downstream signaling pathway of CXCR4) also abolished the gp120-induced α7-nAChR upregulation. *n* = 4 mice/treatment. Results are shown as mean ± SEM values. Matched One-Way ANOVA with Holm-Sidak’s post-test, *P ≤ 0.05.

*In vitro* results have demonstrated that the gp120-induced α7-nAChR upregulation occurs via CXCR4, an HIV coreceptor known to bind gp120^9,21^. To determine whether the α7-nAChR upregulation in our gp120-tgm model occurs through a similar mechanism, WT striatal neurons were exposed to endotoxin-free recombinant gp120_IIIB_ by itself or in combination with AMD3100 (CXCR4 antagonist) and PD98059 (MEK inhibitor) to then measure α7-nAChR expression levels using Bgtx labeling through confocal imaging (Fig. 2). We confirmed that gp120_IIIB_ induces an upregulation of the α7-nAChR in striatal neurons (*P* ≤ 0.05), consistent with results above (Fig. 1A). Moreover, pretreatment with AMD3100 abolished the gp120-induced α7-nAChR upregulation (Fig. 2), suggesting that the mechanism described in Ballester *et al*.^9^, where activation of CXCR4 is needed for α7-nAChR upregulation, is conserved in our gp120-tgm model. Furthermore, pretreatment with a MEK inhibitor (a downstream effector of CXCR4 activation) also abolished the gp120_IIIB_-induced α7-nAChR upregulation (Fig. 2), thus supporting the finding that gp120_IIIB_ effects are mediated through CXCR4. To confirm the region specificity, WT hippocampal neurons were also exposed to gp120_IIIB_ and no α7-nAChR upregulation was observed, consistent with our previous results (Supplemental Figs 1 and 2, and Fig. 1).

### Upregulation of the α7-nAChR leads to higher calcium influx into gp120-tgm striatal neurons

**Figure 3:**
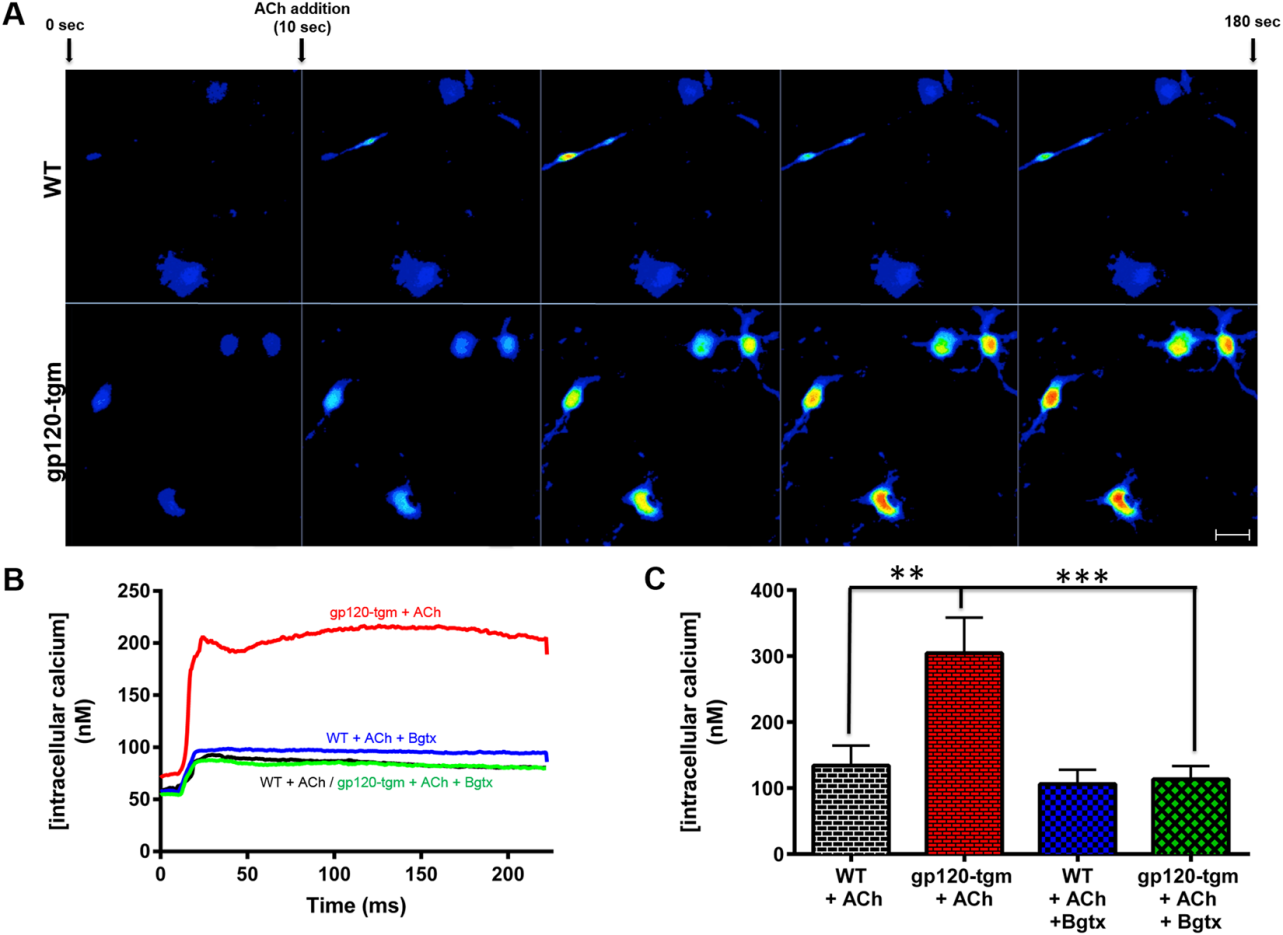
Activation of α7-nAChRs in gp120-tgm striatal neurons leads to higher calcium levels. (**A**) WT and gp120-tgm striatal neurons were incubated with the selective calcium indicator Fluo-4 AM to measure fluorescence levels (which correlate to calcium concentrations). After 10 secs of recording, ACh was added to activate α7-nAChRs, and the recording continued until 180 secs had elapsed. (**B**) Fluorescence intensity was used to calculate calcium concentrations curves for striatal neurons. Red represents striatal neurons from gp120-tgm mice activated with ACh, which show a significant increase in calcium. Green represents gp120-tgm cells with Bgtx pre-treatment. Black represents striatal neurons from WT mice activated with ACh, while blue represents WT cells with Bgtx pre-treatment. (**C**) Quantification of the maximum calcium concentrations indicates that, after activation with ACh, gp120-tgm neurons reach calcium concentrations approximately two times higher (2.27 fold increase) than WT striatal neurons. This effect is blocked by preincubation with the selective antagonist Bgtx, confirming α7-nAChR’s role in calcium curves. Results are shown as mean ± SEM values. One-way ANOVA analysis with Bonferroni’s posttest, *n* = 5 mice/strain, **P ≤ 0.01 ***P ≤ 0.001. Scale bar = 20 μm for all images in panel A.

The α7-nAChR is the most calcium-permeable nicotinic receptor subtype^22^. Taking into account the receptor’s conductive properties and the fact that calcium dysregulation has been associated with neurodegeneration^23^, we measured calcium levels on striatal neurons after activation of the α7-nAChR with its endogenous ligand acetylcholine (ACh). Striatal neurons were loaded with Fluo 4-AM to measure differences in calcium levels within cells. Figure 3A shows that activation of the α7-nAChR with ACh leads to an increase in fluorescence that differs between mice strains. Fluorescence intensities were then converted into calcium concentrations^24^ to generate calcium curves as a function of time. From these curves, we observed that activation of the α7-nAChR results in calcium influx into both WT and gp120-tgm striatal neurons. However, this influx is significantly higher on gp120-tgm striatal neurons, and it is sensitive to Bgtx blockade (Fig. 3B). Furthermore, after averaging maximum intracellular calcium concentrations (Fig. 3C), we found that activation of α7-nAChRs on gp120-tgm striatal neurons leads to more than twice the amount of calcium (WT neurons 134.3 nM vs. gp120-tgm neurons 304.5 nM; ≈ 2.3 fold increase; *P* ≤ 0.01), suggesting that gp120 induces an increase of functional calcium-conducting receptors. Furthermore, blockade of α7-nAChRs with Bgtx significantly reduces calcium levels comparable to WT cells (WT neurons 106.0 nM vs. gp120-tgm neurons 113.5 nM), confirming the receptor’s participation in the increased calcium levels. These results demonstrate that: i) gp120 exposure leads to an increase in the quantity of functional receptors, ii) activation of the α7-nAChR significantly increases intracellular calcium concentrations on gp120-tgm striatal neurons, and iii) blockade of α7-nAChRs reduces intracellular calcium concentrations to control levels in gp120-tgm striatal neurons.

### Gp120-tgm exhibit higher apoptotic activity on striatal neurons and behavioral impairments on a striatum-dependent paradigm

HIV-gp120 induces the upregulation of functional α7-nAChRs, followed by a significant increase in calcium influx into gp120-tgm striatal neurons (Fig. 3C). Calcium ions are key to various cellular processes ranging from synaptic activity to cell-cell communication. However, disruption of normal levels can lead to neurodegenerative diseases^23^. We investigated whether the increased calcium influx into gp120-tgm striatal neurons, as a consequence of α7-nAChR upregulation and activation, could be sufficient to drive striatal neurons to apoptosis. To this end, we co-labeled striatal neurons with Annexin-V and propidium iodide (PI), markers of apoptosis, to quantify the percentage of cells undergoing apoptosis. Incubation of gp120-tgm striatal neurons with ACh significantly increased the percentage of apoptotic cells when compared with cells under basal conditions (14.6% gp120-tgm without ACh vs. 27.7% gp120-tgm with ACh; *P* ≤ 0.05), suggesting that activation of the α7-nAChR is, in fact, sufficient to drive cells to apoptosis. To confirm the contribution of the α7-nAChR on this percentage increase, cells were pre-treated with Bgtx prior to ACh addition. Blockade of the α7-nAChR significantly decreases the percentage of apoptotic cells (27.7% gp120-tgm with ACh vs. 14.3% gp120-tgm with Bgtx pretreatment; *P* ≤ 0.05), demonstrating the role of α7-nAChRs in the observed increase in apoptosis. On WT striatal neurons, there were no statistical differences across treatments, a finding that is expected since activation of the α7-nAChR is necessary for the normal functioning of different cells, including neurons^25,26^. These results suggest that the upregulation of α7-nAChRs (as consequence of gp120 presence) and subsequent calcium entry after α7-nAChR activation could contribute to the gp120-induced neurotoxicity and cell death in the striatum.

**Figure 4:**
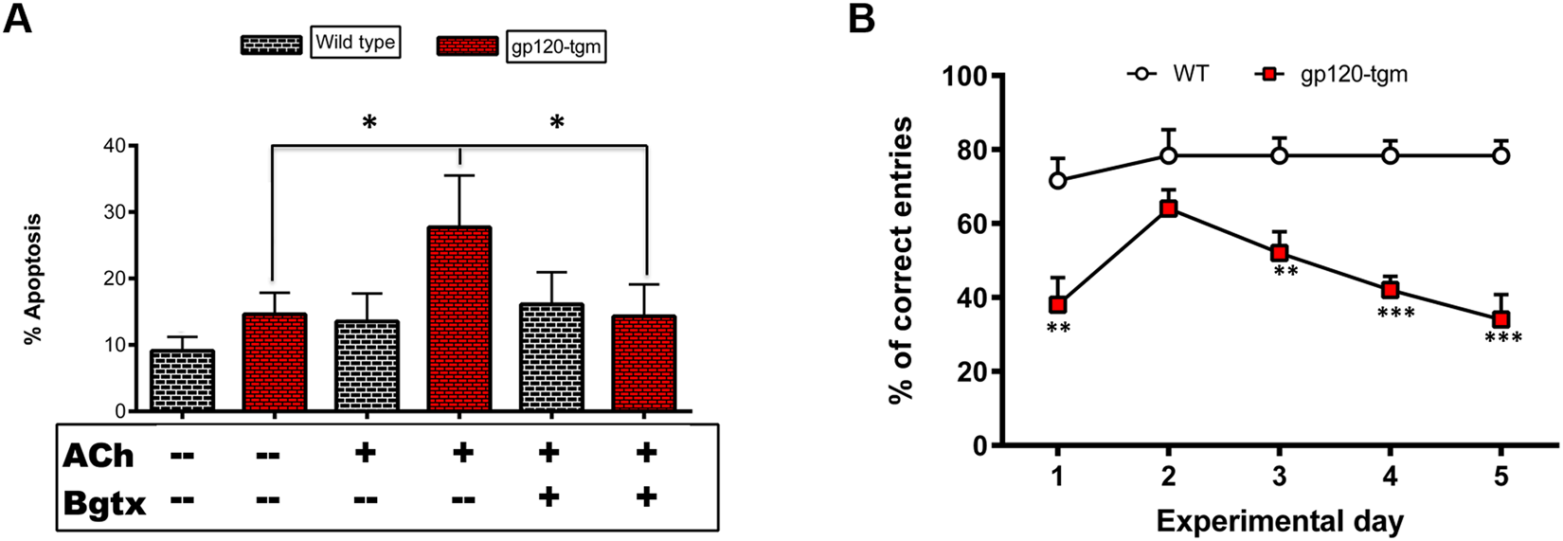
Activation of α7-nAChR in gp120-tgm striatal neurons leads to apoptosis. Striatal neurons from WT, and gp120-tgm mice were incubated with ACh (1 hr) to activate α7-nAChR or with a pretreatment of Bgtx (30 min) and then ACh (1 hr) to block α7-nAChR function. Cells were labeled with Annexin-V and Propidium Iodide (PI) to measure those undergoing apoptosis processes. (**A**) The percentage of striatal neurons labeled with Annexin-V and PI was quantified. White bars represent results for WT striatal neurons, red for gp120-tgm. Activation of α7-nAChR with ACh exacerbated the condition on gp120-tgm striatal neurons where the percentage of apoptotic cells doubles, an effect attenuated by Bgtx preincubation. (**B**) In a separate experiment, WT and gp120-tgm were trained to perform a delayed non-matching (DNM) paradigm, to assess striatum-dependent behavioral differences. The experimental phase lasted for 5 days. Since day one, except for day two, gp120-tgm consistently performed poorer than WT mice, suggesting striatal-dependent cognitive impairment as early as 4-months of age. Results are shown as mean ± SEM values. Two-Way ANOVA for apoptosis assays, *n* = *5* mice/strain, *P ≤ 0.05, scale bar = 20 μm. Two-Way ANOVA for behavioral test, *n* = *6* for WT and *n* = *5* for gp120-tgm, **P ≤ 0.001, ***P ≤ 0.0001.

HAND has been associated with neurological problems including gliosis, inflammation, striatal pathology, and cognitive decline^27,28^, presumably due to the large accumulation of gp120 in human’s basal ganglia^14^. Based on this, we aimed to determine if gp120-tgm showed striatum-dependent learning and behavioral deficiencies. WT and gp120-tgm were trained using a delayed non-matching (DNM) paradigm, which is designed to test spatial working memory^29^. Results show that gp120-tgm exhibited a poorer performance than WT mice that continued to decrease as the experiment progressed (Fig. 4B; **P ≤ 0.01, ***P ≤ 0.001). These results suggest that the presence of gp120 in the striatum promotes cognitive impairments as early as four months of age.

### Decreased locomotor activity in the gp120-tgm model

One of the neurologic problems associated with HAND is motor dysfunction^2^. Because the striatum is one of the brain areas involved in locomotion^30^ and it is the region where the gp120-induced α7-nAChR upregulation takes place, we measured voluntary wheel running in an experimental setup previously validated by our group^31,32^. Both WT and gp120-tgm mice locomotor activity was assessed over a seven-day period where daily activity was recorded. Total traveled distance, total time spent on the wheel, number of events, and event time duration were measured and averaged for each experimental group (saline, bupropion, and MLA-treated mice; Table 1). Results show that on average, saline-treated gp120-tgm run less distance and spend less time on the wheel (Table 1), in comparison to saline-treated WT mice. When looking at the events, both mice strains have comparable numbers, but event duration is lower for gp120-tgm (Table 1). Overall, gp120-tgm demonstrate a poorer locomotive performance than their WT counterpart. Treatment with bupropion, an FDA-approved, smoking cessation, antidepressant drug previously used on HIV^+^ patients^33–35^ and a noncompetitive antagonist of α7-nAChRs^18^ seems to improve gp120-tgm performance, but has no major effects on WT mice (Table 1). Accordingly, in WT mice, antagonizing α7-nAChRs with MLA seems to worsen locomotive performance on the wheel, whereas it tends to improve gp120-tgm performance.

**Table 1:** Gross locomotor performance of WT and gp120-tgm with different treatments.

Strain	Treatment	Distance (m)	Total time (min)	Events (#)	Event time (min)
WT	Saline	2789	162	309	58
	MLA	1441	117	232	54
	Bupropion	2640	152	334	47
Gp120-tgm	Saline	881	66	263	13
	MLA	2574	93	418	26
	Bupropion	1439	104	498	16

**Figure 5:**
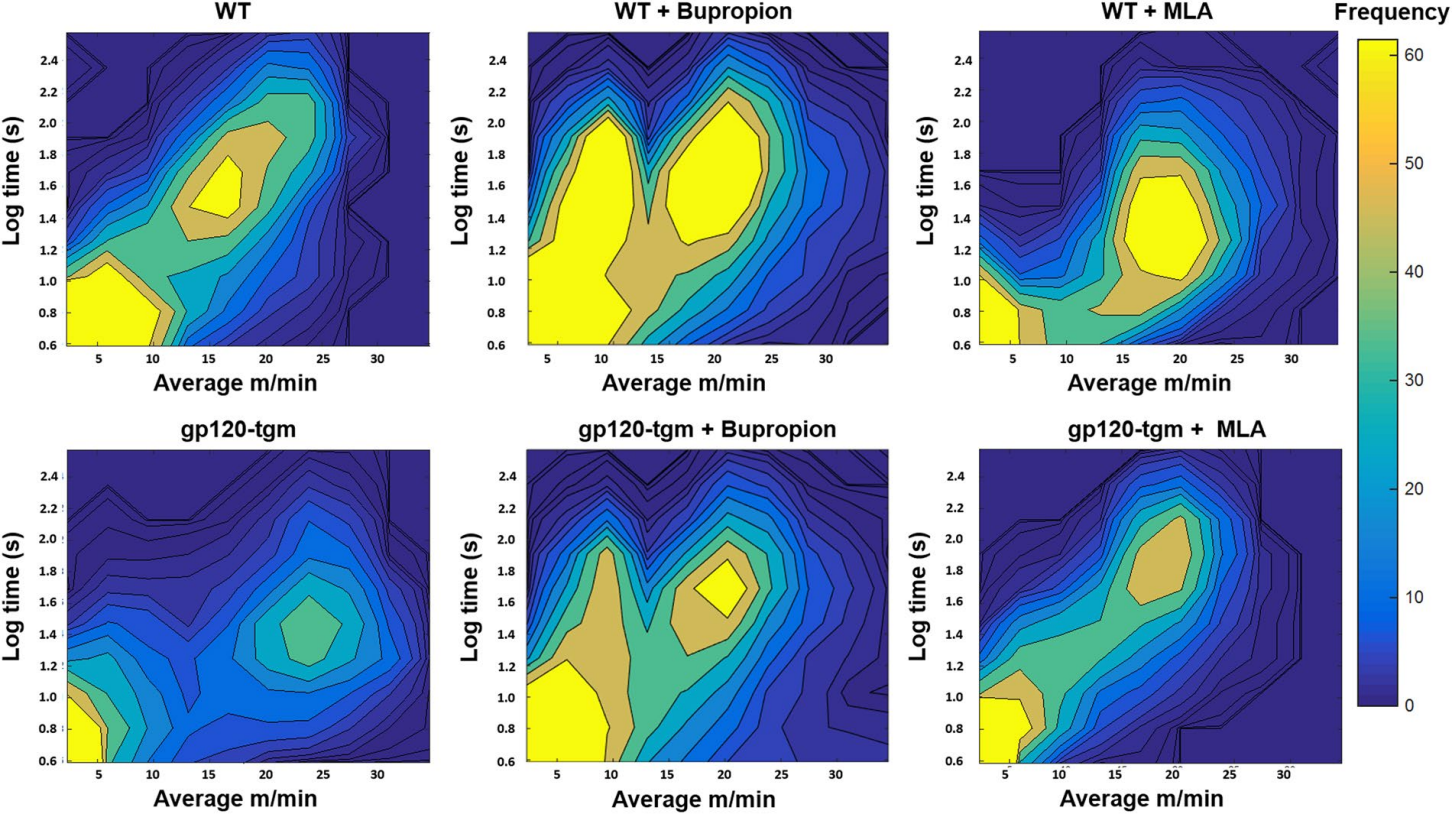
HIV-gp120-tgm exhibit hindered locomotor activity, which can be improved by antagonizing the α7-nAChR. Locomotor activity of WT and gp120-tgm under different conditions (saline, bupropion, MLA) was measured over a seven-day interval. Time spent running, and their corresponding velocities were plotted on heat maps in order to determine periods of activity burst of each treatment group. Results demonstrate that gp120-tgm engage in physical activity but for shorter time periods, seen as a decrease in the frequency of high activity periods. Moreover, treatment with α7-nAChR antagonists increases the frequency of these high activity periods and overall activity. *n* = 6 for Saline-treated and Bupropion-treated WT mice; *n* = 5 for Bupropion-treated gp120-tgm; *n* = 3 for Saline-treated gp120 mice and MLA-treated WT and gp120-tgm.

This type of analysis provides a general scenario of mice locomotor activity. However, because many factors come into play in locomotion, we cannot say with certainty, whether the differences seen between WT and gp120-tgm are due to locomotor impairments or simply to behavioral differences across strains (for instance, gp120-tgm could be less motivated to use the activity wheel, hence the differences). To this end, we analyzed the same data to generate heat maps and examine in detail the frequency of activity bursts (activity bursts of a given velocity for a defined period). With this type of analysis we can segregate voluntary wheel running into a collection of time points, detect more subtle differences, and observe locomotive patterns^32^. The data presented in Fig. 5 represent the running activity profile of these mice, and it permits us to observe two distinct peaks: i) activity periods of low velocity and duration (lower left quadrant) and ii) activity for longer periods of time (Y-axis) at higher velocities (X-axis). Notably, results show that the ability of gp120-tgm to run for extended periods is compromised (activity peak is less intense, therefore less frequent), suggesting that the presence of gp120 stimulates a notably negative effect on striatal-associated functions such as locomotor activity. It stands out that MLA, an antagonist of α7-nAChRs, increases the frequency of this activity peak (Fig. 5), suggesting an improvement in locomotor performance as a result of receptor antagonism. Moreover, MLA treatment also changes the duration and speed of these events (seen as an upward shift in the high activity peak). We found locomotor improvements on gp120-tgm that are consistent with the results seen in Table 1, but with this analysis (heat maps), we dissected the differences in more detail. Interestingly, bupropion also increases the ability of gp120-tgm to run for extended periods of time at high velocities, a treatment strategy that could be further explored since bupropion is an already used drug for HIV^+^ patients^33,34^. Overall, our results demonstrate that gp120 presence is sufficient to impair the locomotor activity of gp120-tgm since a young age and also suggest that antagonizing the α7-nAChR, either by bupropion or MLA treatment, improves performance in these animals.

## Discussion

During development of HAND, several brain regions are affected, and regional differences in patterns of neuronal damage have been reported in autopsy studies^36^. In fact, the basal ganglia is compromised earlier in the illness^15,17^ while other areas, such as the cortex and hippocampus, at later stages^13^. It is known that the basal ganglia contain cholinergic neurons and interneurons that express α7-nAChRs^37,38^. Moreover, neuronal populations vary in their susceptibility to HIV-mediated damage based, in part, on the expression levels of chemokine receptors^36^ such as CXCR4. In this study, we demonstrate that the α7-nAChR is upregulated on neurons from the striatum of gp120-tgm (Fig. 1) and that this upregulation is mediated by CXCR4 (Fig. 2). CXCR4 is recognized and activated by gp120_IIIB_, the same glycoprotein expressed in our gp120-tgm^6^. These results are consistent with previous reports demonstrating that gp120 is able to promote cell death in a CXCR4-dependent fashion^9,39–41^.

The mouse model used in this study is a valuable tool to study gp120-induced neurotoxicity and molecular pathways associated with gp120 exposure; however, it has its limitations. We only assess the contribution of gp120, when it has been demonstrated that other viral proteins have significant effects in the CNS^42–45^. Thus, we cannot rule out the contribution of other viral proteins in the pathogenesis of HAND, and we cannot exclude the possibility that, in the presence of intact virions, other mechanisms could attenuate or enhance the contribution of α7-nAChRs. Furthermore, this is a non-inducible model, therefore, neurons are exposed to gp120 throughout development, which could alter their susceptibility to the glycoprotein^3^. However, by having only one viral constituent in our transgenic model, we can dissect the specific contribution of gp120 in HIV-induced neurocognitive disorders and its effects on α7-nAChR expression in a unique, isolated, and controlled environment, even though it may not fully recapitulate what is happening *in vivo*.

The function and properties of α7-nAChRs in an HIV setting depend on its expression patterns. For example, we recently demonstrated a dysregulation of α7-nAChR gene expression in postmortem brain samples recovered from HIV-infected subjects at different stages of HAND^11^. Moreover, a recent study determined that α7-nAChR upregulation (by gp120) could be detrimental to brain endothelial functions and that it could increase amyloid beta transport across the brain^46^. Conversely, in our study, we did not observe an increase in the expression of α7-nAChRs in microglia, although this upregulation has been demonstrated in monocyte-derived macrophages^10^. Microglia plays a pivotal role in HAND^47–49^ and it has been demonstrated that they regulate inflammation through the same mechanisms as macrophages, through the cholinergic anti-inflammatory pathway, where α7-nAChR is an essential player^50,51^. Thus, the α7-nAChR is an important target for neurocognitive disorders, however, its expression on different cell types (neurons or microglia) could define the receptor’s role and contribution to disease development and progression.

Previous studies have proposed the upregulation of α7-nAChR as the cell’s response to desensitization^52^ to compensate for decreased receptor activity. However, our scenario differs from this one. In our experimental setting, the upregulation of α7-nAChR occurs through activation of CXCR4 and not as the cell’s compensation mechanism. As evidenced by our calcium results (Fig. 3), α7-nAChRs present on gp120-tgm are functional and responsible for the ACh-evoked calcium influx. These findings are consistent with another study where they demonstrate that nicotine is able to restore gene expression after being altered by HIV expression. Specifically, in the dorsal striatum, the authors show that one of the pathways restored by nicotine is calcium signaling^28^. A specific nicotinic receptor subtype is not implicated, but these results strengthen the results of our study. Furthermore, we also demonstrate that this increase in calcium influx is sufficient to drive neurons to apoptosis (Fig. 4A). This finding is consistent with a study demonstrating that sustained opening of α7-nAChRs with positive allosteric modulators can result in intracellular calcium overloading, only when the α7-nAChR is overexpressed in the cell membrane^53^. Hence, we can support the findings of Ballester *et al*.^9^, where activation of α7-nAChR leads to neuronal death/apoptosis, thus positioning the α7-nAChR as a novel pharmacological candidate to prevent or ameliorate gp120-induced neurotoxicity.

Previous studies on the gp120-tgm found no cognitive impairments in young mice (3-month old mice) but on older mice (10-month old mice)^8^. It is important to notice that this study used a water maze, a hippocampus-dependent task. Because it has been shown that on HAND the hippocampus is affected but subcortical structures, such as the striatum, are first and most severely affected^4,54,55^, we aimed to determine if gp120-tgm had striatum-dependent learning and behavioral deficiencies. We assessed mice performance on a delayed non-matching paradigm, which focuses on spatial working memory and is affected by ventral striatum lesions^29^. This paradigm helped demonstrate striatum-dependent learning differences between WT and gp120-tgm, since gp120-tgm performed significantly poorer than WT mice (Fig. 4B), suggesting cognitive impairment in these animals. Assessing cognitive deficiencies using a striatum-dependent instead of a hippocampal-dependent task could explain why we observed cognitive decline as early as 4-months.

Locomotor activity has been previously employed in other studies to assess psychomotor function in rodents and is a model previously validated by our group^31,32^, even though it has not been studied in this transgenic model. Several aspects of voluntary wheel running have been examined (genetic, environmental, etc.) in the literature and some studies suggest that it is a motivated activity strongly influenced by striatal dopamine^56–59^. Indeed, locomotor activation provides an indirect index of increased dopamine release in the striatum^60^. Interestingly, α7-nAChR function has also been associated to the striatum, and it has been shown that it can modulate dopamine and glutamate function^61,62^. Furthermore, it has been associated with the nigrostriatal pathway, particularly in the basal ganglia motor loop^62^. In fact, the basal ganglia contain cholinergic neurons and interneurons^37,38^, therefore, a role for this receptor on locomotor activity is evident. As measured, gross locomotor function is decreased in gp120-tgm (Table 1). However, measurements of total distance, total time, events, and event time, although informative, do not represent the complexity of this activity. Several factors such as motivation, muscular weakness, among others, come into play in wheel running. This is the reason for the more detailed analysis described in Fig. 5 in addition to the one-dimensional analysis (Table 1). Heat maps were constructed to demonstrate the activity profile of mice and make a more specific comparison of the gp120-tgm locomotor abilities with their WT counterparts. From Fig. 5, we observe that gp120-tgm are engaging in physical activity but abandon it after short periods of time, leading to decreased frequency of high-activity periods. Therefore, in our transgenic model, psychomotor performance is affected by the presence of gp120.

As aforementioned, the gp120-induced α7-nAChR upregulation leads to neuronal apoptosis; therefore, it is conceivable to propose that antagonizing the receptor could have potential benefits. As seen in Fig. 5, antagonizing the receptor improves gp120-tgm locomotive performance, since both bupropion and MLA treatment increased the number of events and the frequency of high activity periods on gp120-tgm. Bupropion acts as an antagonist for α7-nAChRs^18^, however it is not selective. It can also act as an antagonist for dopamine reuptake inhibitors, α4β2, and α3β2 nicotinic receptors^18^; therefore, it cannot be discarded that the improvement seen in gp120-tgm, as a result of bupropion treatment, could be due to other mechanisms and not to α7-nAChR antagonism. Regardless of its selectivity, bupropion treatment resulted in an improvement of locomotor performance in gp120-tgm. Because this is an already used drug for HIV^+^ patients^33,34^, these results open the possibility for the use of this treatment to improve and/or ameliorate the symptoms in these patients. On the other hand, MLA, which is a highly selective antagonist of α7-nAChR, also improves gp120-tgm performance but gave an opposing result in WT mice. In the gp120-tgm, where the α7-nAChR is upregulated, antagonizing the receptor decreases its functionality, and possibly calcium influx, thus normalizing cell function and preventing apoptosis, giving a more marked improvement on gp120-tgm performance. Conversely, antagonizing the receptor in WT mice lowers receptor functionality, impairing locomotor performance. These results could suggest that variations in α7-nAChR basal levels affect locomotor performance in mice.

Overall, the present study expands and complements the results obtained by Ballester *et al*.^9^. Moreover, our results are also consistent with the deregulation of α7-nAChR expression levels in post-mortem basal ganglia samples recovered from HIV-infected subjects exhibiting different degrees of HAND demonstrated by Ramos-Busot *et al*.^11^. These studies are proof of the detrimental consequences of the chronic presence of HIV constituents, such as gp120, in the destruction of striatal neurons and the neurocognitive deterioration experienced by HAND patients. Furthermore, although these two previous works and ours focus on the cellular and molecular events that occur in the CNS, there is also evidence in Delgado-Vélez *et al*.^10^ of peripheral alterations (upregulation) in the expression of α7-nAChR in MDMs exposed to gp120_IIIB_. It appears that deregulation of α7-nAChR expression levels is part of the cellular alterations experienced by striatal neurons, MDMs, monocytes, and T-lymphocytes in HIV-infected patients. More importantly, the simultaneity of α7-nAChR expression alterations at the central and peripheral levels outlines the complexity of the disease as well as the challenge for pharmacological intervention. The specific and fundamental roles of α7-nAChRs on normal cognition processes and its functional versatility make it an important pharmacological target to be considered in HIV-related complications. Here we provide a novel and unexplored approach to better understand HIV-induced cognitive decline.

## Materials and Methods

### Animals

The University of Puerto Rico (Rio Piedras Campus) Institutional Animal Care and Use Committee (IACUC) approved the protocol for these studies (IACUC#: R1-02002-08-11-2014). All animal studies were conducted following institutional guidelines for animals use. Adult male transgenic mice expressing HIV-1 gp120 in their brains under the regulatory control of a modified murine glial fibrillary acidic protein (GFAP) gene were derived from Lennart Mucke’s line (described in ref.^6^) and obtained from Jackson Laboratories. Wild type (WT) mice were also obtained from Jackson Laboratories as a B6SJLF strain (a cross between C57BL/6 J female x SJL/J male) and used as the genetic background for gp120-tgm mice. Animals were housed in clear plastic ventilated cages, maintained in a temperature- and humidity-controlled room on a 12-h light/dark cycle with food and water provided *ad libitum*. Mice were used for experimentation once they reached 4-months of age, after verifying gp120 expression by genotyping. Genotyping was performed using the KAPA HotStart Mouse Genotyping kit (KAPA Biosystems, Wilmington, MA) following manufacturer’s instructions.

### Isolation of mouse adult neurons and microglia

Isolation of mouse adult neurons and microglia was performed following the protocol described by Brewer and Torricelli, 2007^63^. Briefly, mice were sacrificed by cervical dislocation, their brains removed and plated over ice. Mice’s striatum and hippocampus were dissected manually using stereotaxic coordinates taken from the Paxinos and Franklin mouse brain atlas^64^, and combined in Hibernate A (BrainBits LLC, Springfield, IL), 2% B27 supplement (Invitrogen, Eugene, OR), and 0.5 mM Glutamax (Invitrogen, Eugene, OR) for 8 min shaking at 200 rpm and 30 °C. Tissue was then digested using 2 mg/mL papain (Worthington, Lakewood, NJ), diluted in Hibernate A minus calcium (BrainBits LLC, Springfield, IL) for 30 min at 30 °C. After digestion, tissue was transferred to 5 mL of Hibernate A media where it was allowed to come to room temperature. Then, tissue was triturated with 9″ Pasteur pipettes coated with a silanization solution (Sigma-Aldrich, St. Louis, MO), and the supernatants collected. The supernatant was then added to an Optiprep (Sigma-Aldrich, St. Louis, MO) density gradient, where neurons were separated from debris. From the four layers obtained from the gradient, the top 7 mL layers of debris were discarded and layers 1–3 were recovered (a mixture of neurons and microglia). Viable cells were identified and counted by Trypan blue exclusion assay. Cells were then plated on 15 mm glass coverslips (for Bgtx binding assays) (Warner Instruments, Hamden, CT) or on 4-well tissue culture slides (for calcium measurements and apoptosis experiments) (Nalgene Nunc International, Rochester, NY) coated overnight with poly-D-lysine (Sigma-Aldrich, St. Louis, MO), 100 μg/mL in distilled water. Neurons were cultured in media containing the following composition: Neurobasal A/B27/Glutamax (Invitrogen, Eugene, OR) with 10 ng/mL FGF2 and 10 ng/mL PGDFbb (Invitrogen, Eugene, OR) for trophic support. Cells were cultured for 7–9 days at 37 °C in 5% CO_2_, 95% O_2_ with media change at days 2 and 5. Microglia were cultured in media with the following composition: Neurobasal A (Invitrogen, Eugene, OR) supplemented with 20% horse serum (Invitrogen, Eugene, OR) and the media changed at days 2 and 5.

### Treatment with gp120_IIIB_, AMD3100, or PD98059

Striatal neurons from 4-month old male WT mice were isolated as described above and cultured for 7–9 days in Neurobasal A/B27/Glutamax (Invitrogen, Eugene, OR) with 10 ng/mL FGF2 and 10 ng/mL PGDFbb (Invitrogen, Eugene, OR). Then, cells were pretreated for 30 min with either AMD3100 (0.1 μM) or PD98059 (10 μM) and subsequently incubated with gp120_IIIB_ for 24 hrs. After 24 hrs, a co-treatment of gp120 _IIIB_/AMD3100 or gp120 _IIIB_/PD98059 was given for an additional 24 hrs (in new media). Finally, cells were fixed and incubated with Bgtx as described below.

### α-bungarotoxin binding assay

Primary neuronal and microglial cultures grown in 15 mm glass coverslips were fixed by incubation with 4% formaldehyde (15 minutes), followed by incubation with 1:100 Bgtx-Alexa-555 (to label α7, Invitrogen, Eugene, OR) and 1:500 CD90-FITC (to label neurons, Becton Dickinson, CA) or CD80-PE (to label microglia, Becton Dickinson, CA) for 1hr at room temperature in dark. Cells were then washed 3 times with PBS 1X(pH 7.2), coverslips placed with mounting media containing DAPI (to label cellular nuclei), and fluorescence acquired by confocal imaging (Zeiss LSM Meta 510; Carl Zeiss, Pleasanton, CA) at the Confocal Imaging Facility, University of Puerto Rico (http://cifupr.org/) under a 40X magnification.

### Intracellular calcium measurements

For calcium measurements, cell culture media was changed to Hibernate A media (BrainBits LLC, Springfield, IL) to then incubate with Fluo-4 AM 10 μM for 45 minutes in the dark. Neuronal cultures were then pretreated with Bgtx 100 nM (to block α7) for 30 min. Then, pyridostigmine 1 mM (to inhibit acetylcholinesterase activity) was added for 10 min (both after Fluo-4 AM incubation) prior to Acetylcholine (ACh) addition. Cells were then washed with PBS 1X (pH 7.2) and again placed in Hibernate A (in the dark) to start recordings. ACh 1 mM was added to the media (after 10 sec of recording), to activate α7-nAChR, on cells previously incubated with Bgtx or without pretreatment, while being excited at a wavelength of 488 nm using an Argon/2 laser. Emission was acquired using a BP 500–550 filter on a Zeiss LSM 510 META confocal microscope (40X). Images were acquired in a time series of 200 msec. Cytoplasmic Ca^2+^ concentrations were determined using the “F_max_” equation^24^: [*Ca*^2+^] = *K*_*d*_*[*(*F/F*_max_ − *1/R*_*f*_)*/*(*1* − *F/F*_max_)] where K_d_ is the dissociation constant (345 nM) of Fluo-4 AM^65^, *F* is the mean fluorescence value, *F*_max_ is the Fluo-4 AM fluorescence at saturating Ca^2+^ concentrations and R_*f*_ is Fluo-4’s dynamic range (*F*_max_/*F*_min_) previously determined as 100^24,65^.

### Apoptosis assay

Striatal neurons were placed on Hibernate A media and pretreated with Bgtx 100 nM for 30 min. Neuronal cultures were then treated with ACh 1 mM for 1 hr and washed with PBS 1X (pH 7.2). Binding to Annexin-V and Propidium Iodide (PI) was performed using the CytoGLO Annexin-V-FITC Apoptosis Detection Kit (eBioscience, San Diego, CA) with minor modifications. Briefly, after incubation with Bgtx and/or ACh, cells were washed with PBS 1X(pH 7.2) and placed in the binding buffer (1X). Then, cells were labeled with both Annexin-V and PI (1:50) for 25 min in the dark. Binding to Annexin-V and PI was visualized using confocal imaging (Zeiss LSM Meta 510) under a magnification of 40X.

### Radial arm maze delayed nonmatching behavioral assay

Male WT and gp120-tgm (4-month old) were individually housed and trained to perform a recurring choice, delayed nonmatching (DNM) paradigm using an eight-arm radial maze. Initially, mice were acclimated to the environment of the maze (by exploring the maze) and the handlers (daily holding for 3 consecutive days, 10 min/day). Additionally, mice were food deprived to stimulate the search of a reward (Fruit Loop®). The paradigm used the same three arms on all trials, 90° to the left and to the right of the holding arm (T-shape configuration). Trials consisted of a sample phase (training) and a test phase (testing). In the sample phase (10 days-10 sessions/day/animal), two arms were opened: (1) the holding arm, and (2) one of the two choice arms (randomly selected). Mice were trained to travel from the holding arm to one choice arm and back to the holding arm. Once the mice returned to the holding arm, the gate was closed to retain the mice for the duration of the retention interval (3 secs). Once the retention interval was over, the gates to the three arms were opened, and reinforcement was placed on the arm not previously entered. Mice were expected to travel to this arm and back to the holding arm, to begin the next trial. After training, the test phase (5 days-10 sessions/day/animal) was performed using the same configuration as the sample phase. Responses were scored as correct when mice entered the arm where reinforcement was available without re-entering the previously reinforced arm.

### Locomotor activity measurements

Male WT and gp120-transgenic mice (4-month old) were trained and habituated on an activity wheel system. Daily drug intraperitoneal (i.p.) injections started on the habituation/training period and continued throughout the testing period. Bupropion (Sigma-Aldrich, St. Louis, MO) 40 mg/kg and methyllycaconitine (MLA) 3.4 mg/kg (Sigma-Aldrich, St. Louis, MO) (doses that have proven to be both physiologically correct and tested in other studies^66,67^) were prepared and administered in a sterile saline solution (0.9% NaCl). After the training period, mice were kept for one week in computer-monitored, clear polycarbonate cages containing wheels (diameter 12.7 cm) that recorded average velocity (meters/minute) and total distance (meters) traveled at a frequency of 1 Hz on a 24 hr-period (wheel counter model 86061, USB computer interface model 86056A, activity wheel monitor software version 9.2, Lafayette Instruments, Lafayette, IN). Daily recordings were obtained to calculate total distance traveled, number of events (times the mouse got on the wheel), duration and frequency of events. Subsequently, the data was analyzed to obtain a graph of average velocity as a function of time, in other words, to determine periods of activity bursts. The frequency of each activity burst was laid on a scatter plot to generate a heat map (with MatLab), which gave us information about the ability of mice to run for a certain period of time at a specific velocity.

### Statistical Analyses

A *P* ≤ 0.05 was considered to be significant. All statistical analyses were performed with GraphPad Prism 6 (GraphPad, San Diego, CA).

### Data Availability

No datasets were generated or analyzed during the current study.

## Electronic supplementary material

Supplemental Figures
