# Supplementary material for: The alpha7-nicotinic receptor contributes to gp120-induced neurotoxicity: implications in HIV-associated neurocognitive disorders
Source: Sci Rep. 2018 Jan 29;8:1829. doi: 10.1038/s41598-018-20271-x (PMC5788855; doi:10.1038/s41598-018-20271-x)
Supplement: Supplementary file 1 — Supplemental Figures [file 41598_2018_20271_MOESM1_ESM.doc]

The alpha7-nicotinic receptor contributes to gp120-induced neurotoxicity: implications in HIV-associated neurocognitive disorders

Coral M. Capó-Vélez^1,4^, Bryan Morales-Vargas^1^, Aurian García-González^1^, José G. Grajales-Reyes^1^, Manuel Delgado-Vélez^1,4^, Bismark Madera^1,4^, Carlos A. Báez-Pagán^2^, Orestes Quesada^2^, José A. Lasalde-Dominicci^1,3,4^

^1^University of Puerto Rico, Río Piedras Campus, Department of Biology, San Juan, P.R. 00931-3360

^2^University of Puerto Rico, Río Piedras Campus, Department of Physical Sciences, San Juan, P.R. 00931-3360

^3^University of Puerto Rico, Río Piedras Campus, Department of Chemistry, San Juan, P.R. 00931-3360

^4^University of Puerto Rico, Molecular Sciences and Research Center, San Juan, P.R. 00926

Corresponding author:

José A. Lasalde-Dominicci, Ph.D.

jlasalde@gmail.com

Supplemental Information

*
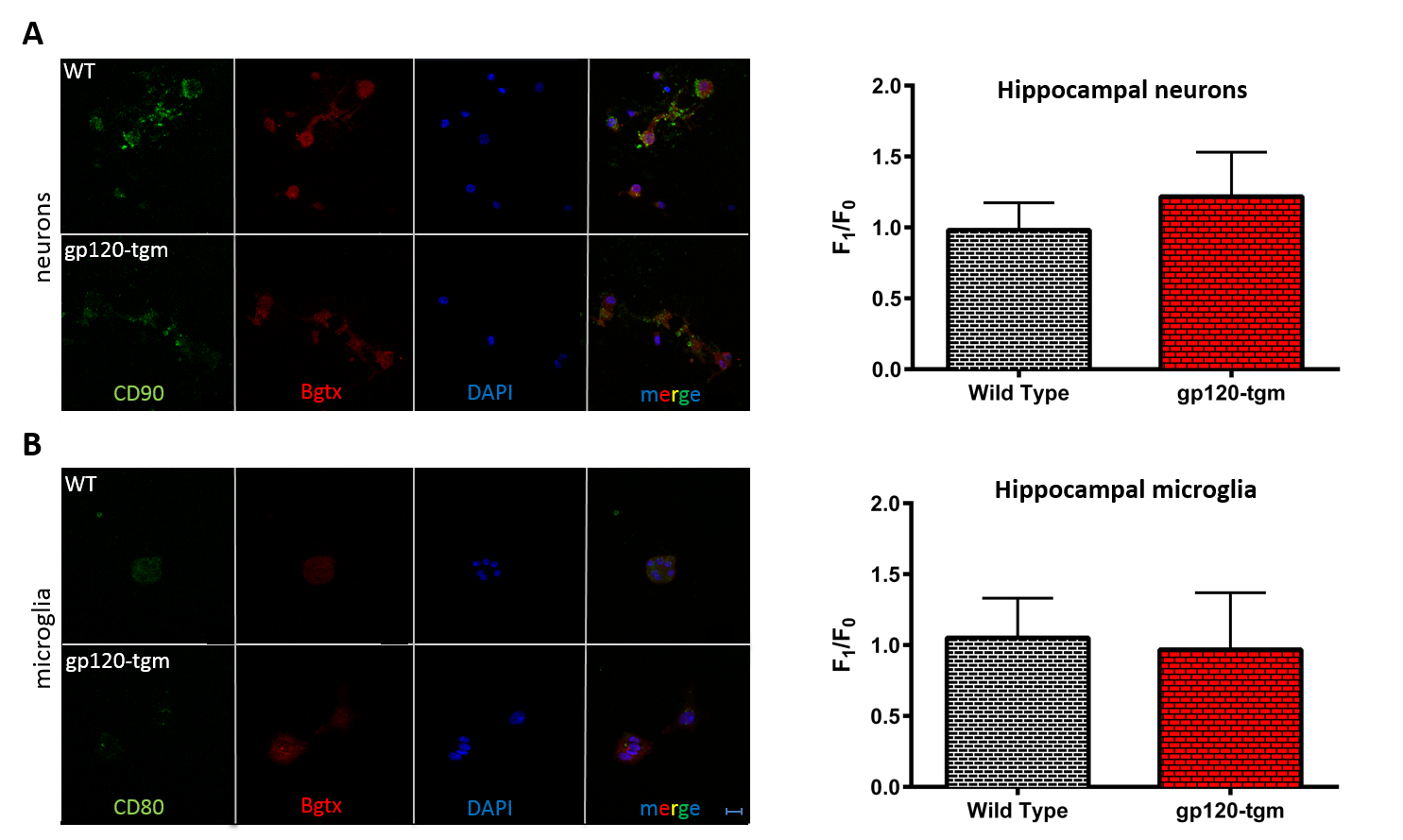
*

***Supplemental Figure 1****. Neither neurons nor microglia from gp120-tgm hippocampus exhibit α7-nAChR upregulation.* Hippocampal neurons and microglia were isolated from WT and gp120-tgm adult mice. After culture (7-9 days), cells were fixed with formaldehyde 4% and incubated with (**A**) CD90 (neuronal marker – green) or (**B**) CD80 (microglia marker - green), Bgtx (α7-nAChR marker – red), and DAPI (nucleus marker – blue) for examination through confocal imaging (40x). After relative quantification, it is evident that neither neurons (*n* = 5 mouse/strain) nor microglia ( *n*= 3 mouse/strain) from gp120-tgm exhibit significant changes in α7-nAChR expression when compared to WT cells, suggesting that gp120 does not have an effect on α7-nAChR expression in this brain region. Unpaired student’s t-test, Scale bar = 40 μm.

*
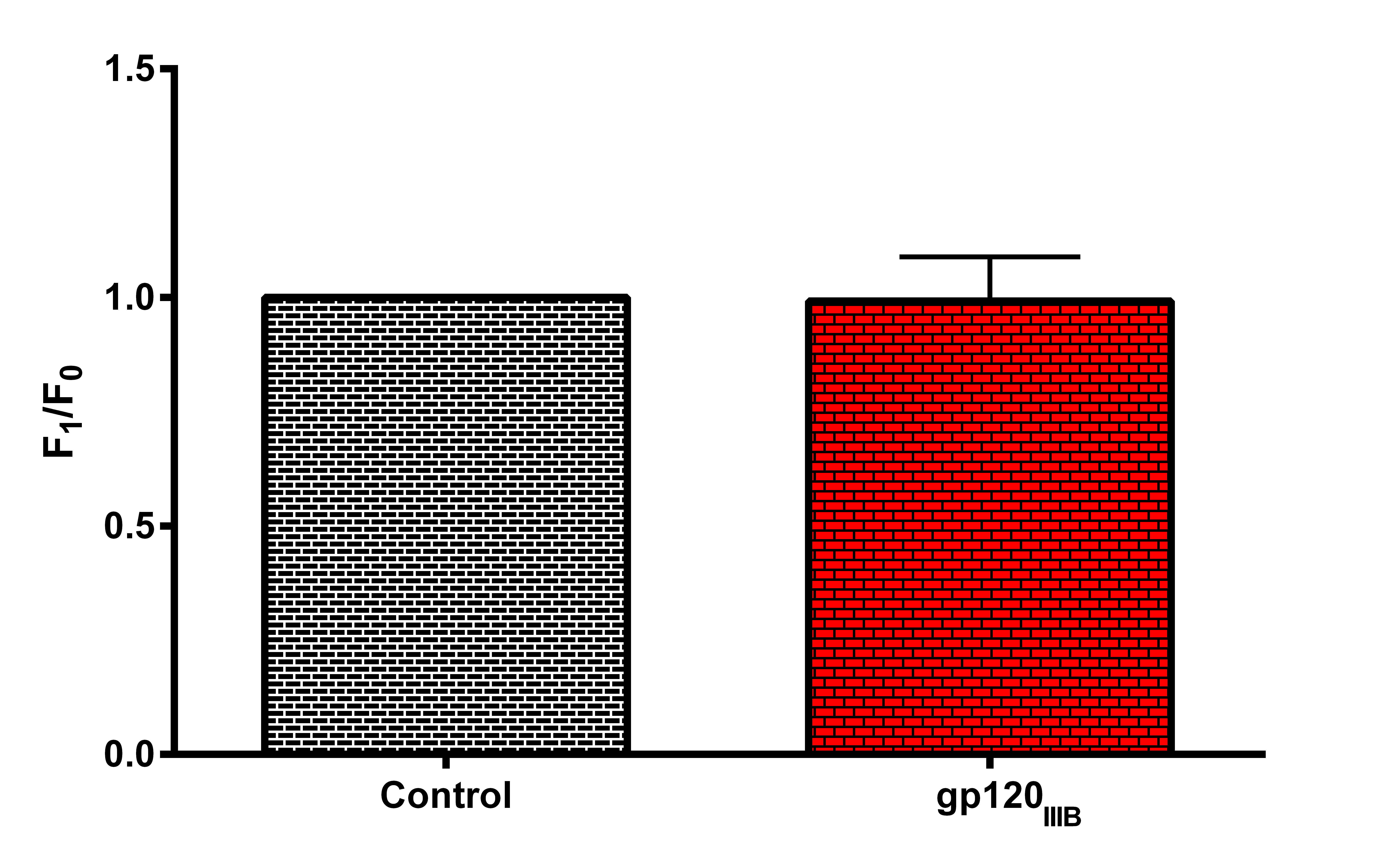
*

***Supplemental Figure 2.*** *The gp120-induced upregulation of α7-nAChR is not seen in hippocampal neurons.* Hippocampal neurons were isolated from WT mice and treated with gp120_IIIB_ for 48hrs. Cells were then incubated with Bgtx and DAPI to measure α7-nAChR levels by confocal imaging. Results show that exogenous gp120_IIIB_ is not able to upregulate α7-nAChRs. *n=*4 mice/treatment. Results are shown as mean ± SEM values. Student’s paired t-test.
